# A double-hit pre-eclampsia model results in sex-specific growth restriction patterns

**DOI:** 10.1242/dmm.035980

**Published:** 2019-02-08

**Authors:** Violeta Stojanovska, Dorieke J. Dijkstra, Rebekka Vogtmann, Alexandra Gellhaus, Sicco A. Scherjon, Torsten Plösch

**Affiliations:** 1 Department of Obstetrics and Gynecology, University Medical Center Groningen, University of Groningen, 9700RB Groningen, The Netherlands; 2 Department of Gynecology and Obstetrics, University Hospital Duisburg-Essen, 45147 Essen, Germany

**Keywords:** Developmental programming, Metabolomics, Pre-eclampsia

## Abstract

Pre-eclampsia is a multifactorial pregnancy-associated disorder characterized by angiogenic dysbalance and systemic inflammation; however, animal models that combine these two pathophysiological conditions are missing. Here, we introduce a novel double-hit pre-eclampsia mouse model that mimics the complex multifactorial conditions present during pre-eclampsia and allows for the investigation of early consequences for the fetus. Adenoviral overexpression of soluble fms-like tyrosine kinase (sFlt-1) and lipopolysaccharide (LPS) administration at mid-gestation in pregnant mice resulted in hypertension and albuminuria comparable to that of the manifestation in humans. A metabolomics analysis revealed that pre-eclamptic dams have increased plasma concentrations of phosphadytilcholines. The fetuses of both sexes were growth restricted; however, in males a brain-sparing effect was seen as compensation for this growth restriction. According to the plasma metabolomics, male fetuses showed changes in amino acid metabolism, while female fetuses showed pronounced alterations in lipid metabolism. Our results show that combined exposure to sFlt-1 and LPS mimics the clinical symptoms of pre-eclampsia and affects fetal growth in a sex-specific manner, with accompanying metabolome changes.

## Abstract (precis)

**Summary:** Double-hit exposure to the anti-angiogenic factor soluble fms-like tyrosine kinase and the pro-inflammatory endotoxin lipopolysaccharide in mice provides a novel, comprehensive model of pre-eclampsia, which leads to sex-specific metabolomic differences in fetuses.

## INTRODUCTION

Pre-eclampsia is a multisystemic pregnancy-associated disorder that is identified after the 20th week of gestation with the onset of hypertension and proteinuria (Mol et al., 2016). More importantly, it is one of the most frequent complications of pregnancy, affecting 3-7% of the population (Mol et al., 2016; Rajakumar et al., 2005). In up to 60% of cases, especially in early-onset pre-eclampsia, it is further complicated by fetal growth restriction (Weiler et al., 2011; Xiao et al., 2003). Moreover, pre-eclampsia and the subsequent fetal growth restriction leads to increased susceptibility of the offspring to chronic cardiometabolic diseases later in life (see Stojanovska et al., 2016 for review). In recent years, it has become apparent that pre-eclampsia shares characteristics with metabolic syndrome, at least through altered angiogenic and inflammatory markers (Salzer et al., 2015; Scioscia, 2017).

Two of the most pronounced pathophysiological mechanisms during pre-eclampsia are the disrupted angiogenic balance and the increased systemic inflammatory responses. First, the concentrations of anti-angiogenic factors are elevated in pre-eclamptic patients (Hertig et al., 2004); for example, elevated levels of circulating soluble fms-like tyrosine kinase 1 (sFlt-1) have been shown to be clearly associated with the severity of pre-eclamptic symptoms (Park et al., 2005). Second, several markers of inflammation, such as tumor necrosis factor alpha (TNFα), interleukin 6 and C-reactive protein, are also increased in the plasma of pre-eclamptic patients (Borzychowski et al., 2006). Moreover, inflammation affects blood pressure and renal function during pregnancy, contributing to the clinical course of pre-eclampsia (Cotechini et al., 2014; Kalinderis et al., 2011).

*In vivo* models of pre-eclampsia are of extreme importance in clarifying the pathophysiological aspects of the disease and the evaluation of potential fetal programming mechanisms. Inflammatory models of pre-eclampsia, such as low-dose endotoxin infusion (Faas et al., 1994) or TNFα administration (Cotechini et al., 2014), have provided significant insights into kidney and placental pathophysiology during pre-eclampsia based on inflammatory mechanisms. Furthermore, models that involve antagonism of angiogenesis (Maynard et al., 2003; Venkatesha et al., 2006) have been widely studied in the evaluation of maternal and fetal health (Bytautiene et al., 2013, 2011; Lu et al., 2007; Patten et al., 2012). These models are each based only on a single pathophysiological mechanism (McCarthy, Kingdom et al., 2011; Sunderland et al., 2011) that results in some of the clinical symptoms of pre-eclampsia, not covering the full pathophysiological spectrum that occurs during this disorder in human patients. Therefore, we aimed to develop a model that involves the interplay of both anti-angiogenesis and inflammation.

During pregnancy, perturbations in maternal health can lead to morphological and functional changes of different organ systems in the offspring, leading to a demonstrable impact on the offspring's health for a lifetime (Davis et al., 2012; Kajantie et al., 2009), an effect known as developmental programming. For example, during early-onset pre-eclampsia, there is a 2- to 4-fold increased risk of fetal growth restriction (Xiao et al., 2003). Putative predisposing factors for the development of cardiometabolic diseases in the growth-restricted offspring include alterations in metabolism, the epigenome or fetal autonomic regulation (Ching et al., 2015; Jiménez-Chillarón et al., 2012; Schäffer et al., 2009). However, far too little attention has been paid to the effects of pre-eclampsia on the maternal and fetal metabolome, which can provide a better understanding of the relationship between early-life circumstances and later-life disease susceptibility at a metabolomics level. The current study describes a novel, double-hit model of pre-eclampsia that closely resembles the complete clinical course. We apply this novel model to investigate the genuine role of anti-angiogenesis and inflammation in pregnant mice with regard to metabolic fetal outcomes and function.

## RESULTS

### Combined sFlt-1 and lipopolysaccharide exposure induces pre-eclampsia symptoms in pregnant dams

**Fig. 1.**
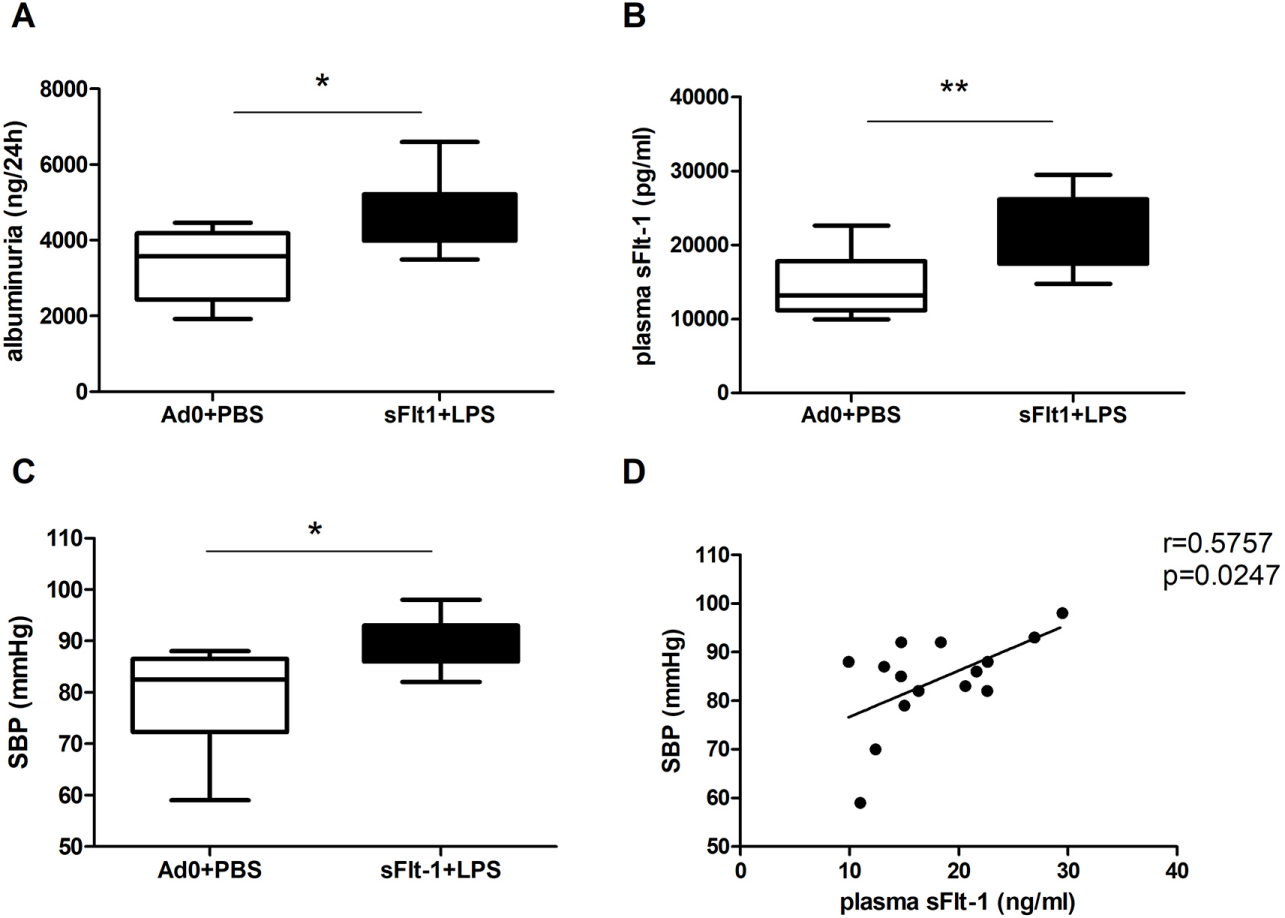
**Double-hit exposure to sFlt-1 and LPS in pregnant dams can induce pre-eclampsia symptoms.** (A) Urine albumin concentration in 24 h urine samples from pregnant dams at GD 17.5 (Ad0+PBS *n*=8; sFlt-1+PBS *n*=10). (B) Plasma sFlt-1 concentrations from pregnant dams at GD 18.5 (Ad0+PBS *n*=9; sFlt-1+PBS *n*=8). (C) Systolic blood pressure (SBP) in pregnant dams at GD 18.5 (Ad0+PBS *n*=8; sFlt-1+PBS *n*=7). (D) Correlation between sFlt-1 plasma concentrations and SBP in pregnant dams (*r*=0.57, *P*=0.02; *n*=15). Data are presented as median and interquartile ranges (A-C; *P<0.05, **P<0.01).

C57Bl/6J mice were subjected to adenoviral overexpression of sFlt-1 and, 48 h later, challenged with lipopolysaccharide (LPS). Weight gain and food and water consumption on gestational day (GD) 17.5 were not different between the groups (Fig. S1B-D). At GD 17.5, total urinary protein excretion (Fig. S1A) and mouse-specific albuminuria (Fig. 1A) were significantly increased in the dams that had been exposed to the double hit of sFlt-1 and LPS. In continuation, these dams had 2-fold increased sFlt-1 concentrations in their plasma (Fig. 1B). On GD 18.5, systolic blood pressure in the pregnant dams exposed to the double hit was also significantly increased in comparison to that in controls (Fig. 1C). Moreover, there was a positive correlation between the blood pressure values and the obtained sFlt-1 concentrations of the pregnant dams (Fig. 1D). There were no differences in the number of pups, nor in the percentage of fetal resorptions between the groups (Fig. S1E,F). Together, these data show that the mid-gestation double-hit exposure to sFlt-1 and LPS replicates the clinical features of human pre-eclampsia in pregnant dams.

### Double-hit pre-eclampsia is not accompanied by changes in placental compartment area

**Fig. 2.**
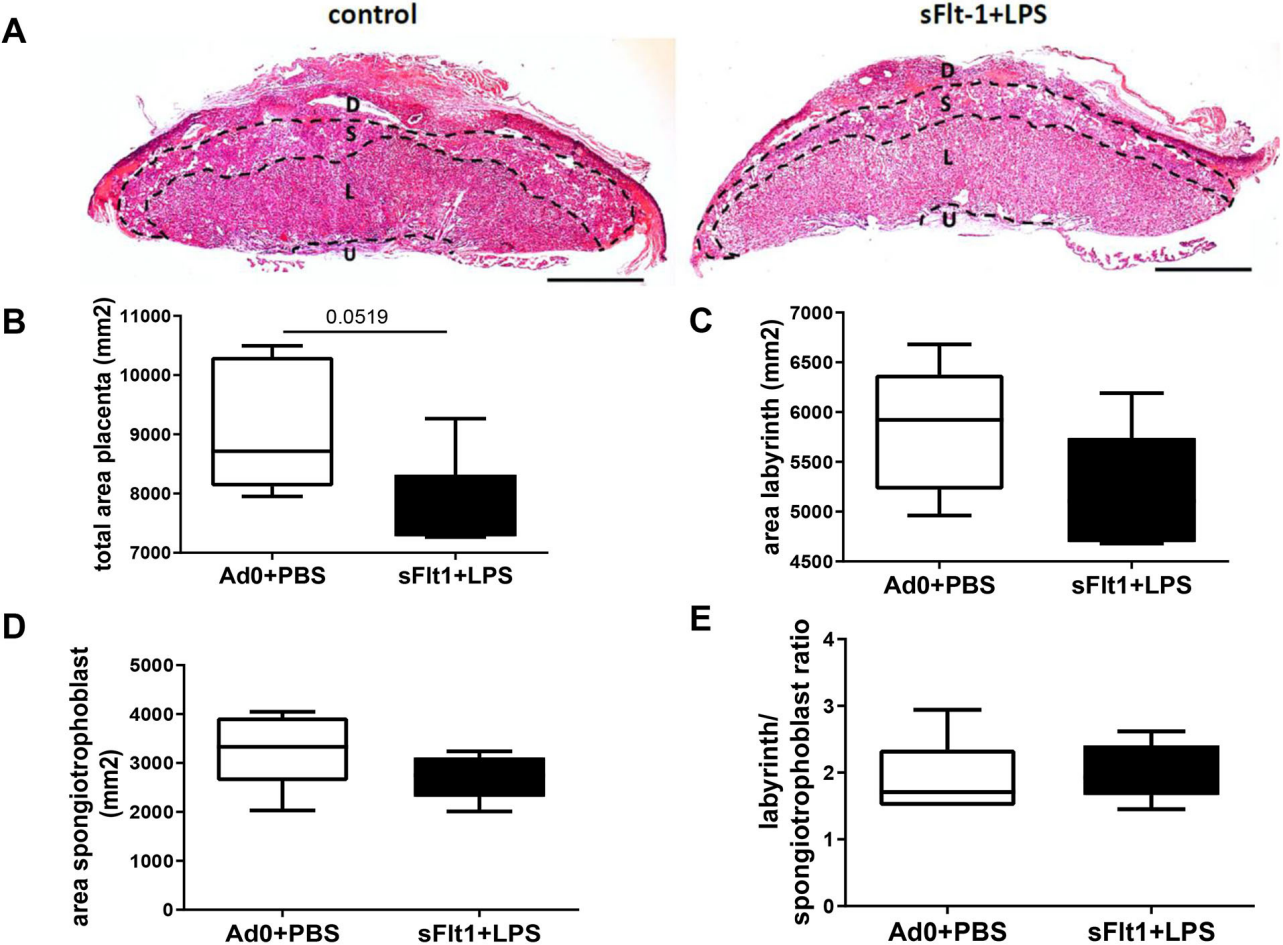
**Placental morphology at GD 18.5 in the double-hit pre-eclampsia model.** (A) Placentae were collected and 7 µm sections were stained with H&E. Scale bars: 1 mm. D, decidua; L, labyrinth layer; S, spongiotrophoblast layer; U, umbilical cord. (B-D) The surface areas of the whole placenta (B), the labyrinth layer (C) and the spongiotrophoblast layer (D) were measured in mm^2^. (E) The ratio of the labyrinth area to the spongiotrophoblast area. Data are presented as median and interquartile ranges (B-E; Ad0+PBS *n*=5; sFlt-1+PBS *n*=6; *P*=0.0519).

In order to evaluate whether the double-hit exposure of pregnant dams to sFlt-1 and LPS differentially affects the placental growth or morphology, we analyzed placental sections at GD 18.5. We assessed total placental area as well as the different placental compartments, namely the labyrinth and the spongiotrophoblast layer. Total placental area tended to be decreased in the double-hit placentae in comparison to that in controls (Fig. 2B, *P*=0.052). This can be attributed both to the labyrinth and the spongiotrophoblast layer, although the specific changes did not reach statistical significance (Fig. 2C,D). Assessment of the labyrinth to spongiotrophoblast ratio showed no differences between the double-hit placentae and the control ones (Fig. 2E). Despite the overall decreased placental area, the placental morphology between the double-hit pre-eclamptic dams and controls was unaffected.

### Maternal plasma phosphatidylcholines are increased during double-hit pre-eclampsia

**Fig. 3.**
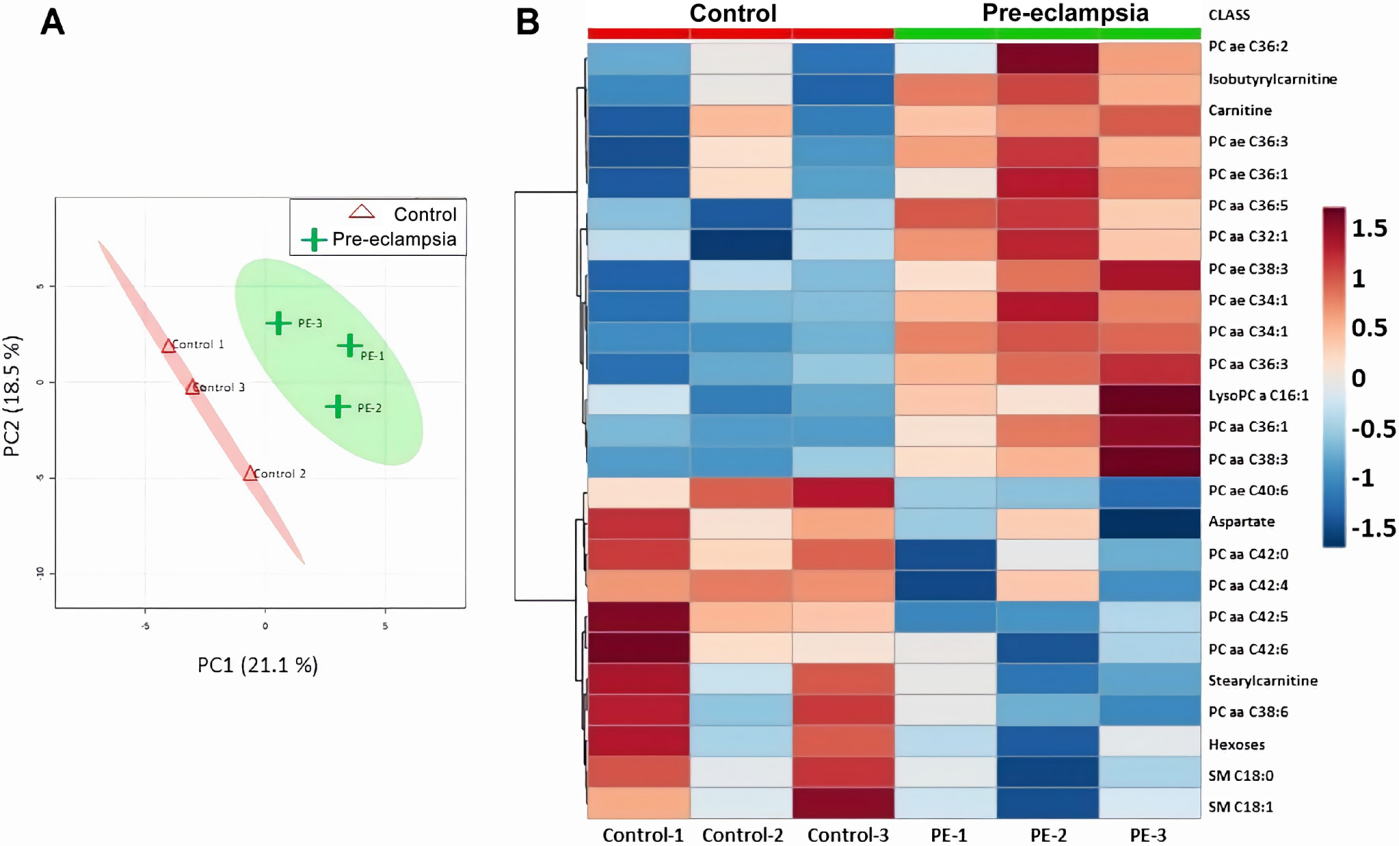
**Maternal metabolome during double-hit pre-eclampsia (*n*=3).** (A) Supervised partial least squares discriminant analysis (PLS-DA) on 141 metabolites in plasma of control and double-hit pre-eclamptic dams (R^2^=0.827, Q^2^=0.348). (B) Heat map representation of the top 25 modified metabolites; color-coding intensity in the red spectrum shows increases in the given metabolites and color intensity in the blue spectrum shows decreases in the given metabolites.

Given that pre-eclampsia is characterized by widespread adaptations in metabolites (Kenny et al., 2010), including altered concentrations of lipids and carnitines (Kelly et al., 2017), we expected that the double-hit pre-eclamptic dams would have a unique metabolomic profile, resembling that of the human condition. A total of 183 metabolites, including monosaccharides, amino acids and several types of lipids (acylcarnitines, sphingolipids and glycerophospholipids), were investigated by tandem mass spectrometry (MS/MS). Metabolites that were below the lower limit of quantification (<LLOQ) were excluded (Table S1); the remaining 141 metabolites were included in the analysis. To identify metabolomic differences between the groups, we performed an unsupervised principal component analysis (PCA) (Fig. S2A) and a supervised partial least squares discriminant analysis (PLS-DA) (Fig. 3A). The results show that the metabolome profile of the double-hit pre-eclamptic dams tends to cluster separately from the one of controls (Fig. 3A). The clear distinction of these groups is based on the variable importance of projection (VIP) scores obtained from each of the 141 metabolites included in the analysis and the top 15 variable compounds are listed in Fig. S2B. A heat map representation of the top 25 modified metabolites showed distinct metabolic differences between the groups, with the levels of a number of metabolites from the class of phosphatidylcholines (PCs) being upregulated in the double-hit pre-eclamptic dams (Fig. 3B). Furthermore, we examined the top modified metabolites with a threshold combination of fold change and *t*-tests. In total, ten metabolites were significantly changed in the plasma from double-hit pre-eclamptic dams, including several long chain fatty acid PCs and acylcarnitine C4 (Table 1).

**Table 1.**
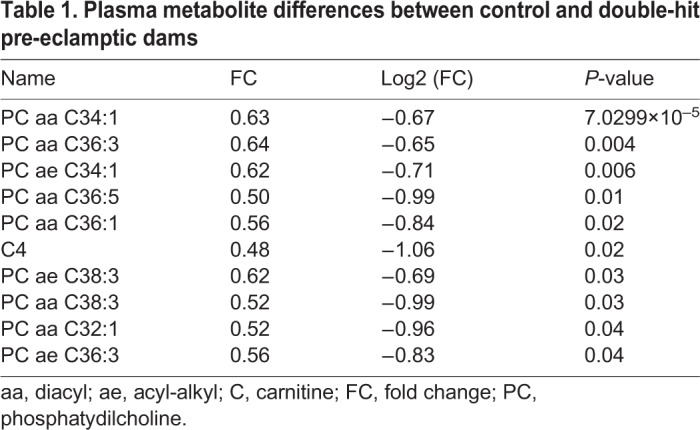
**Plasma metabolite differences between control and double-hit pre-eclamptic dams**

### Fetuses exposed to double-hit pre-eclampsia show growth restriction differences in a sex-specific manner

**Fig. 4.**
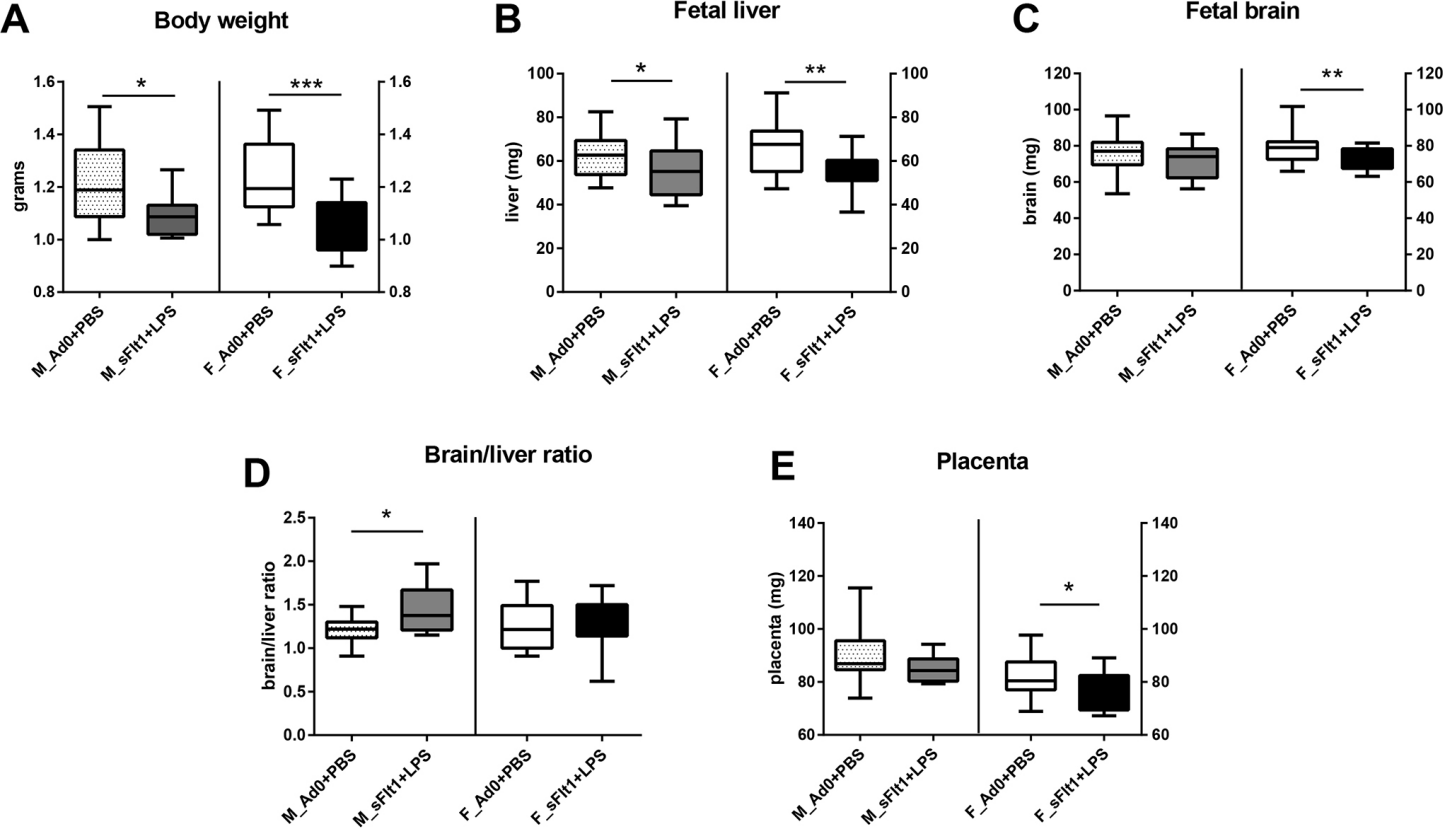
**Fetal characterization at GD 18 in the double-hit pre-eclampsia model.** (A-C) Fetal body weight (A), fetal liver weight (B) and fetal brain weight (C) in grams. (D) Brain-to-liver ratio. (E) Placental weight. Data are presented as median and interquartile ranges (males: Ad0+PBS *n*=17; sFlt-1+PBS *n*=21; females: Ad0+PBS *n*=18; sFlt-1+PBS *n*=19; *P<0.05, **P<0.01, ***P<0.001).

Considering that up to 60% of the early onset pre-eclamptic pregnancies (Weiler et al., 2011) are complicated by fetal growth restriction, we assumed that our double-hit pre-eclampsia model would also lead to impaired fetal growth. Therefore, we phenotyped body size and major organs at GD 18.5 to define the presence, as well as the type, of growth restriction. Male and female fetuses from double-hit pre-eclamptic dams weighed less than fetuses from controls (Fig. 4A). The liver weight was compromised in both sexes (Fig. 4B), while the brain was smaller only in the female fetuses that were exposed to double-hit pre-eclampsia (Fig. 4C). In order to evaluate whether there is a brain-sparing effect in our fetuses, we calculated the brain-to-liver ratio. This was significantly increased for the males, whereas no brain sparing was observed for the females exposed to the double-hit pre-eclampsia (Fig. 4D). These data show that double-hit pre-eclampsia results in fetal growth restriction and that brain sparing is only observed in males.

### The fetal metabolome after double-hit pre-eclampsia exposure shows sex-specific differences

**Fig. 5.**
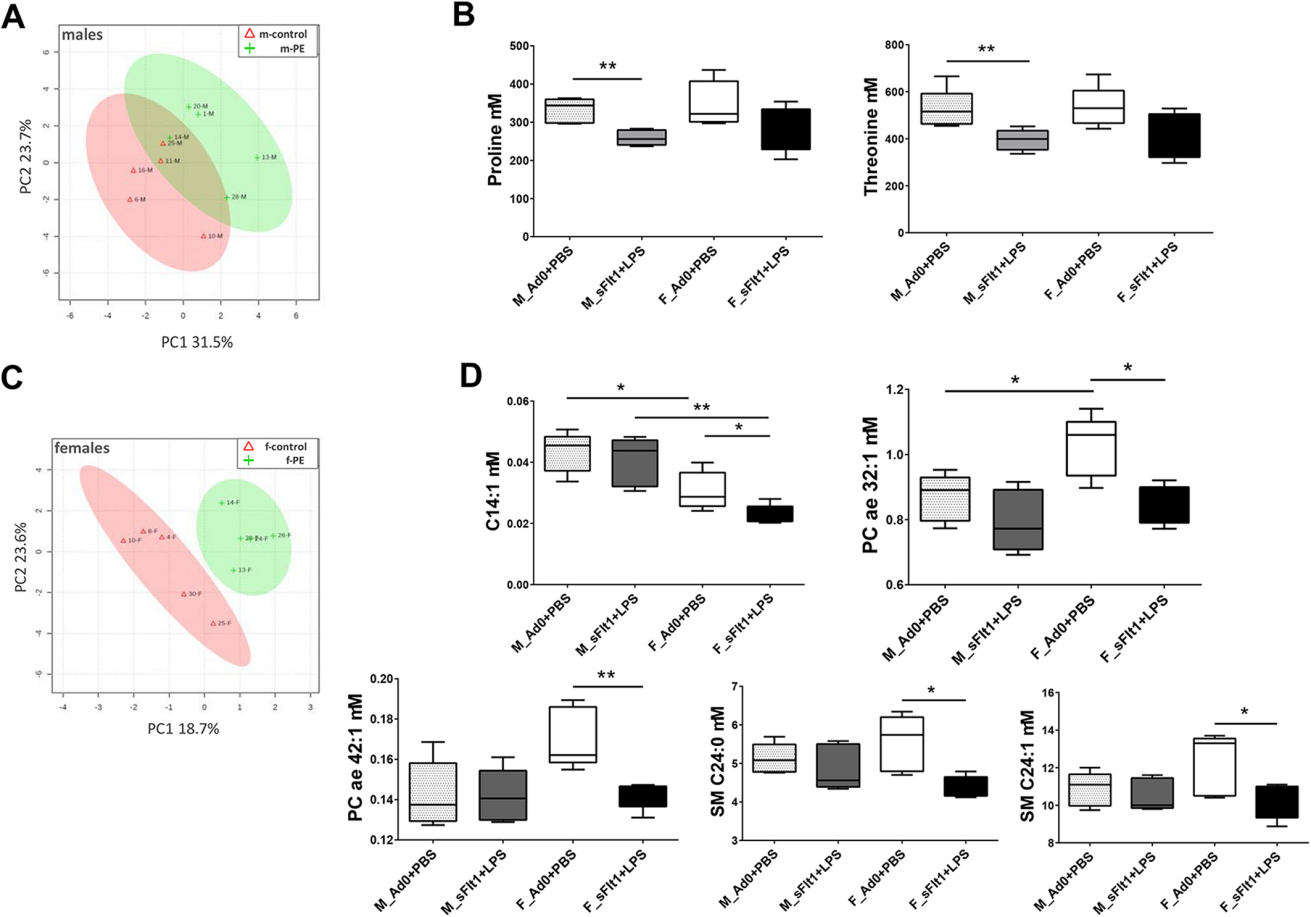
**Fetal metabolomics are differentially** **affected by double-hit pre-eclampsia.** (A) PLS-DA on 141 metabolites in plasma of male control fetuses and double-hit pre-eclamptic male fetuses (R^2^=0.65, Q^2^=0.0607). (B) Proline and threonine concentrations in male and female fetal plasma. (C) Supervised PLS-DA on 141 metabolites in plasma of female control fetuses and double-hit pre-eclamptic female fetuses (R^2^=0.778, Q^2^=0.461). (D) Plasma concentrations of changed metabolites in male and female fetal plasma. Data are presented as median and interquartile ranges (B,D; *n*=5 per group; *P<0.05, **P<0.01).

To explore whether the different growth restriction patterns are associated with metabolomic changes, we analyzed the fetal plasma metabolome. The univariate analysis of log-transformed mouse fetal plasma metabolome data revealed significant sex-specific differences. The unsupervised PCA (Fig. S3A) and the supervised PLS-DA (Fig. 5A) showed an overlap between the metabolic footprint of the males exposed to double-hit pre-eclampsia and the controls. Two metabolites, the amino acids proline and threonine, were significantly decreased in the plasma of the double-hit pre-eclampsia-exposed male fetuses compared with the plasma of controls (*P*<0.05) (Fig. 5B). There were no sex-specific differences between the groups for these metabolites (Fig. 5B). In contrast, the unsupervised multivariate analysis PCA (Fig. S3B) and the supervised PLS-DA (Fig. 5C), revealed a more obvious clustering pattern between the metabolic footprint of female fetuses exposed to double-hit pre-eclampsia and controls. In total, five metabolites showed reduced levels (*P*<0.05) in the plasma from female fetuses exposed to double-hit pre-eclampsia in comparison to controls, including PCs (PC ae 32:1; PC ae 42:1), acylcarnitine (C14:1) and sphingomyelins (SM C24:1; SM C24:0), although only C14:1 and PC ae 32:1 showed sex-specific differences between the control groups (Fig. 5D).

**Fig. 6.**
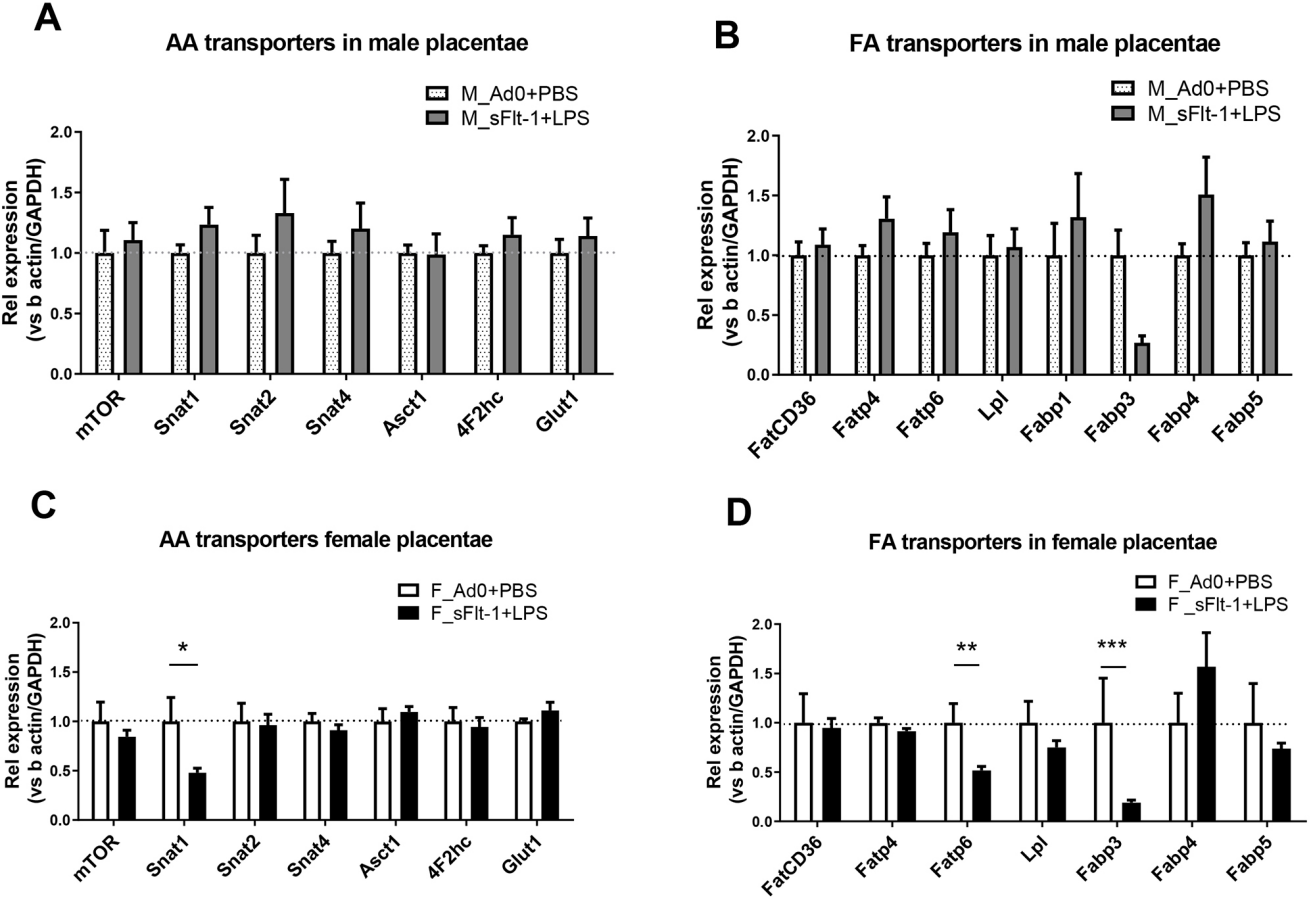
**Gene expression analysis of important placental nutrient transporters.** (A,B) Amino acid (AA) transporters and glucose transporter *Glut-1* (also known as *Slc2a1*) (A) and fatty acid (FA) transporters (B) in male placentae. (C,D) AA transporters and *Glut-1* (C) and FA transporters (D) in female placentae. Data are presented as mean±s.e.m. (Ad0+PBS *n*=8; sFlt-1+PBS *n*=9; *P<0.05, **P<0.01, ***P<0.001).

To determine whether these sex-specific metabolomic differences are potentially associated with changes in placental nutrient transport, we evaluated the expression levels of several amino acid, fatty acid and glucose transporters in the placenta. However, no differences were observed in the gene expression levels between the groups with male placentae (Fig. 6A,B). In contrast, in the female placentae, there was significantly decreased expression of sodium-coupled neutral amino acid transporter 1 (*Snat1*; also known as *Slc38a1*), fatty acid transporter 6 (*Fatp6*; also known as *Slc27a6*) and fatty acid binding protein 3 (*Fabp3*) in the placentae exposed to double-hit pre-eclampsia (Fig. 6C,D).

## DISCUSSION

The results of this study demonstrate that a combined exposure to an anti-angiogenic (sFlt-1) and a pro-inflammatory (LPS) factor lead to the development of pre-eclampsia in mice, mimicking the human clinical course of pre-eclampsia. This double-hit exposure leads to an increase in blood pressure, albuminuria, increased PCs and smaller placentae in the affected dams. Although the placental compartments were not severely compromised, fetuses were growth restricted in a sex-specific manner and showed different metabolomic footprints.

Pre-eclampsia is closely linked to metabolic syndrome on several levels. Obesity and diabetes mellitus serve as known risk factors for pre-eclampsia (Persson et al., 2016; Weissgerber and Mudd, 2015) and increased pro-inflammatory cytokines contribute to the pathogenesis of pre-eclampsia (Cotechini et al., 2014; Lockwood et al., 2008; Pinheiro et al., 2013). Furthermore, pre-eclamptic women are at increased risk of developing cardiovascular diseases later in life (Irgens et al., 2001; Wu et al., 2017). Moreover, dysbalance in angiogenesis affects endothelial function, resulting in changes that resemble pre-eclamptic symptoms, characterized by increased plasma sFlt-1 levels and hypertension (Lu et al., 2007; Maynard et al., 2003; Venkatesha et al., 2006). However, a combined effect of these distinct pathophysiological components to the development of pre-eclampsia has not been addressed thus far. Therefore, a double-hit exposure to anti-angiogenic factors and low-grade inflammation is useful for employing a comprehensive *in vivo* model for pre-eclampsia.

Here, we report that exposure to sFlt-1 and LPS *in vivo* lead to hypertension and albuminuria in the pregnant dam. Although earlier reports suggested that high-dose LPS administration can lead to fetal loss (Kohmura et al., 2000; Silver et al., 1995), inflammation induced by low-dose LPS administration showed no effect on the number of fetuses between the groups in our study (data not shown). sFlt-1 binds to angiogenic factors, such as vascular endothelial growth factors (Vegf proteins) and placental growth factor (Plgf; also known as Pgf), resulting in endothelial dysfunction (Barleon et al., 1997; Tsatsaris et al., 2003). With regard to the impact of endothelial dysfunction on blood pressure, it has been previously shown that inhibition of endothelial protectors (such as eNOS; also known as Nos3) can lead to hypertension (Sander et al., 1999), demonstrating a role for sFlt-1 in blood pressure regulation. In our model, we observed increased plasma sFlt-1 levels to have a positive correlation with blood pressure values. Altogether, we demonstrate that this novel double-hit rodent model is very similar to the human clinical representation of pre-eclampsia.

Studies by Kühnel et al. (2017) using placental-specific overexpression of human sFlt-1 in a lentiviral mouse model of pre-eclampsia, as one hit, led to intrauterine growth restriction in the fetus and resulted in lower placental weights, the same finding as observed in our double-hit model. However, Kühnel et al. (2017) used a smaller labyrinth as the transporting trophoblast, and the loss of glycogen cells in the junctional zone was observed. In contrast to the findings of our study, the expression of the glucose diffusion channel Cx26 was decreased, expression of one fatty acid transporter, CD36, was significantly increased and the amino acid transporters were unchanged in the one-hit model. These differences might be due to the different mouse strains used in the two studies, the continuous sFlt-1 production in the lentivirus model or the lower sFlt-1 concentration in our double-hit model.

Derived from the clinical observation that pre-eclamptic patients have 4- to 8-fold increased risk of developing cardiovascular disorders later in life (Irgens et al., 2001; Wu et al., 2017), characterization of their metabolic footprint is of major interest. In pre-eclampsia, a change in metabolome has been reported (Benton et al., 2016; Kelly et al., 2017), with specific effects on the fatty acid metabolome, sharing similarities with other cardiovascular and idiopathic inflammatory diseases (Famularo et al., 2004; Ruiz-Núñez et al., 2016). Moreover, pre-eclamptic patients show increased choline levels in plasma and urine (Austdal et al., 2014; Friesen et al., 2007), most probably due to increased oxidative stress. In our model, we also report an increase in several types of long-chain fatty acid PCs. Although the metabolic synthesis and function of these PCs has yet to be elucidated, they have been associated with peroxisomal disorders, because peroxisomes are needed for beta-oxidation of long-chain PCs. Moreover, our results are in agreement with the metabolomics analysis of a transgenic model of pre-eclampsia employing catechol-O-methyl transferase knockout mice (Stanley et al., 2015). However, in the model, more profound changes were reported in the metabolome, including increased levels of several PCs, sphingomyelins and acylcarnitines. This can be explained by the different mechanisms applied to induce the pre-eclampsia phenotype, where the catechol-O-methyl transferase knockout acts via the inhibition of enzymes involved in the estrogen conversion. We hence conclude that our joint intervention with sFlt-1 and LPS increases only the glycerophospholipid metabolites without affecting other classes of metabolites.

Exposure to a harsh intrauterine environment has been implicated in sex-specific consequences for the offspring later in life (Lu et al., 2007; Stark et al., 2009). Although the relative contribution of sex on fetal size, body proportions and growth patterns (Melamed et al., 2013) is not well defined, evidence has accumulated that males have increased body weight at birth in comparison to females in uncomplicated pregnancies (Broere-Brown et al., 2016). In addition, in humans, during the first 20 weeks of pregnancy, male fetuses have a greater head circumference in comparison to that of females, but this difference is almost non-existent as the pregnancy proceeds (Broere-Brown et al., 2016). In this context, the timing and exposure to harsh intrauterine stimuli are relevant for sex-specific outcomes. In the current study, we have shown that exposure to sFlt-1 and LPS during mid-gestational days results in smaller brains in female fetuses. In contrast, no weight changes were observed in the male brain, which is consistent with the observation that, in humans, males have decreased growth rate of the head circumference in the last weeks of pregnancy (Broere-Brown et al., 2016), making them then less susceptible to the harsh intrauterine conditions. Data on sex-specific differences in the fetal growth responses due to pre-eclampsia are still limited, but a study from Stark et al. (2009) reported that female infants have significantly lower birth weight percentiles, whereas males maintain normal growth. This is, at least in part, in accordance with our results that female fetuses show symmetrical growth restriction, whereas males show brain sparing and asymmetrical growth restriction.

Sufficient delivery of macronutrients is an important pre-requisite for optimal fetal development. Amino acids, acylcarnitines and glycerophospholipids act as key metabolic factors for the fetus and the placenta (Alexandre-Gouabau et al., 2011). Moreover, a sudden shift in the source of energy will lead to adaptations in several metabolic processes, such as fatty acid oxidation, gluconeogenesis and ketogenesis (Cotter et al., 2013). In response to a hypoglycemic insult, several amino acids, including proline and threonine, serve as gluconeogenic mediators (Houin et al., 2015). Furthermore, an excess of stress hormones (Rando et al., 2016) and inflammatory cytokines (Hashizume et al., 2010) can affect the hepatic lipid catabolism and lipid metabolites severely. In our study, we reported that male and female fetuses are affected with different degrees of growth restriction and have differentially affected metabolic profiles. Whereas the males only have lower concentrations of amino acids such as proline and threonine, the females show decreased levels of certain acylcarnitines, sphingomyelins and glycerophospholipids. This suggests that the symmetrical growth-restricted female fetuses in our double-hit pre-eclampsia model have dysbalanced fat and energy metabolism. Recently, a study reported differences in the cord blood metabolome from pre-eclamptic neonates in comparison to that from controls (Jääskeläinen et al., 2018). The most affected metabolites are similar to the ones we report and included acylcarnitines, PCs and metabolites of urea and tryptophan metabolism. However, the concentrations of these metabolites were higher in the cord blood from pre-eclamptic neonates and there were no clear distinction between the sexes. Moreover, it is not clear whether the collected cord blood was venous or arterial, in order to distinguish between neonatal and maternal background of the plasma. In conclusion, in our double-hit pre-eclamptic model, fetuses show metabolic differences, which are clearly sex specific.

Finally, it is also possible that alterations in transport processes in the placenta contribute to the observed growth-restriction phenotype. As a first step, we here measured the gene expression of several transporters in the placenta. Interestingly, we registered limited changes in the gene expression pattern of nutrient transporters in the placenta, and only decreased levels of amino acid transporter (*Snat1*) and fatty acid transporters (*Fabp3* and *Fatp6*) were observed in females placentae exposed to double-hit pre-eclampsia. In particular, it is known that these fatty acids transporters are increased in obese pregnancies (Díaz et al., 2015), but are quite resilient to hypoxic conditions (Jadoon et al., 2015). Moreover, decreased levels of *Snat1* are associated with growth restriction (Chen et al., 2015; Jansson et al., 2002) and can be correlated with the severity of the restriction. Our findings that these transporters are downregulated only in the female double-hit pre-eclamptic placentae suggest that they might be involved in the mechanisms leading to the growth restriction and metabolic changes. Compatible with this, upregulation of placental transporters may contribute to fetal overgrowth (Jansson et al., 2006; Segura et al., 2017). In contrast, gene expression was not altered in the male placentae, and the minor changes in the metabolic footprint of male fetuses exposed to double-hit pre-eclampsia might be explained by increased fetal or placental consumption of certain metabolites. However, the mechanisms underlying the different metabolomics patterns in fetuses exposed to pre-eclampsia have yet to be fully elucidated, as here we could only determine gene expression levels and not metabolite fluxes.

**Fig. 7.**
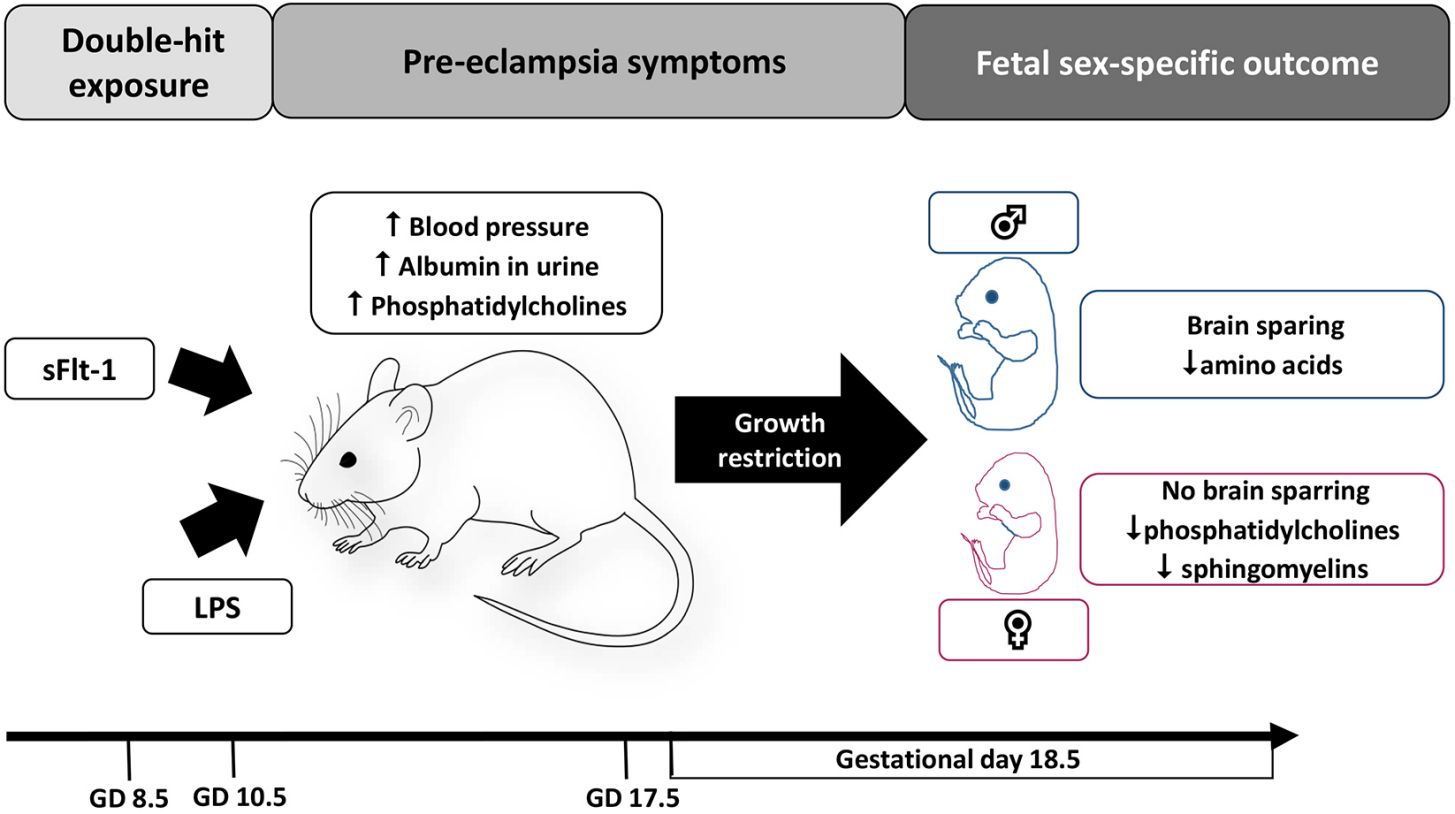
**Maternal and fetal changes due to double-hit experimental pre-eclampsia at different timepoints.**

In conclusion, in this study, we present a clinically relevant mouse model that closely mimics human pre-eclampsia. Moreover, it results in sex-specific differences in the growth restriction pattern and metabolomic footprint (Fig. 7), which in turn can shed light on the sex-specific programming effect of adult-onset disorders due to pre-eclampsia.

## MATERIALS AND METHODS

### Animals and experimental procedures

C57Bl/6J mice (Charles River, France), between 9 and 12 weeks old, were housed in a light- and temperature-controlled facility (lights on from 07:00 until 19:00, 21°C). Mouse chow diet (2186 RMH-B, AB diets) and water were provided to the animals *ad libitum*. Animals were timely mated overnight. When a vaginal plug was present the following day it was counted as GD 0.5. At GD 8.5, animals were randomly assigned to receive either recombinant adenovirus encoding mouse sFlt-1 (Ad-sFlt1) or empty control adenovirus (Ad-null) via retro-orbital injection. At GD 10.5, animals received either 25 µg/kg LPS (*Escherichia coli* 0111:B4, Sigma-Aldrich, St Louis, MO, USA) (the group that received AdsFlt-1) or PBS (the group that received Ad-null). The dosages of adenovirus and LPS have previously been established in pilot studies (data not shown). At GD 16.5, the pregnant animals were placed in a metabolic cage for 24 h to collect urine and measure food and water consumption. At GD 18.5, blood pressure was assessed via the abdominal aorta (Datex-Ohmeda, Cardiocap/5). Placenta and fetal tissues were collected at GD 18.5. Tissue weights were directly recorded as fresh weight. All experiments were approved by the Institutional Animal Care and Use Committee of the University of Groningen (DEC number 6803).

### Amplification and purification of sFlt-1 and control adenovirus

Adenovirus vector stock of Ad-null (a kind gift from U. J. Tietge, University Medical Center Groningen, The Netherlands) and Ad-sFlt1 (a kind gift from S.A. Karumanchi, Beth Israel Deaconess Medical Center, Boston, MA, USA) were used for adenoviral gene delivery. Viruses were amplified in HEK293A cells at a multiplicity of infection of 10. Adenoviral purification was performed with a cesium chloride (CsCl) density gradient (d=1.45 g/ml and 1.20 g/ml). Adenoviral elution was performed with DG columns (Bio-Rad, Temse, Belgium). The concentration of plaque forming units (PFU) was analyzed with an enzyme-linked immunoassay that detects the adenoviral hexon (Adeasy viral titer kit, Agilent Technologies, Santa Clara, CA, USA); 1×10^9^ PFU of adenovirus expressing an empty vector (Ad-null; *n*=9) or mouse sFlt-1 (Ad-sFlt-1; *n*=9) in 100 µl PBS were injected via the retro-orbital plexus on GD 8.5.

### Plasma analysis

Maternal blood was collected on GD 18.5 in EDTA-containing tubes (Greiner Bio-One, Kremsmünster, Austria) with a heart puncture. Within 30 min, the blood was centrifuged for 20 min at 1000 ***g*** and the plasma was stored at −80°C until analysis. Fetal blood was collected by nicking the left ventricle of the heart while the fetuses were slightly tilted, in order to keep the pooled blood in the thoracic cavity while it was collected in EDTA-coated capillary tubes (Greiner Bio-One). sFlt-1 concentrations in plasma were determined using a mouse sFlt-1 ELISA kit (R&D Systems, Minneapolis, MN, USA) according to the manufacturer's protocol.

### Plasma metabolome detection

Plasma was obtained and stored as described above. Plasma metabolome analysis was performed with a Biocrates AbsoluteIDQ p180 Kit at their facility (Biocrates Life Sciences AG, Innsbruck, Austria), as described previously (Stanley et al., 2015). In short, a commercially available direct flow injection and liquid chromatography (LC)-MS/MS kit was used to analyze 188 available metabolites in plasma samples, including hexose (1), amino acids (21), biogenic amines (21), glycerophospholipids (90), sphingolipids (15) and acylcarnitines (40). Internal standards were pre-pipetted, and a calibration standard mix in seven different concentrations was included in a standardized assay in 96-well plate format. Per sample, 10 µl plasma was loaded in each well. A Waters Acquity BEH C8 column (75 mm×2.1 mm, particle size of 1.7 μm) (Waters, Milford, CT, USA) was used for chromatographic separation at 50°C using a gradient mixture of solvent A (water with 0.2% formic acid) and solvent B (acetonitrile with 0.2% formic acid) at a flow rate of 0.9 ml/min using a linear gradient. The optimized parameters included capillary at 3.2 kV, desolvation gas flow at 1200 l/h, cone gas flow at 150 l/h, desolvation temperature at 650°C, source temperature at 150°C and cone voltage at 10V. The samples were delivered to an API4000 Qtrap^®^ tandem mass spectrometry instrument (Applied Biosystems, Foster City, CA, USA), using a reverse-phase high-performance LC column followed by a direct flow injection assay.

### Urine analysis

Urine samples were collected by placing the pregnant dams in metabolic cages at GD 16.5 for 24 h. The protein and albumin levels were determined using a Pierce BSA Protein Assay Kit (Thermo Fisher Scientific, Waltham, MA, USA) or Assaypro Mouse Albumin ELISA kit (St. Charles, MO, USA), respectively. The concentration of total protein and albumin per sample was multiplied by the 24-h urine volume.

### Tissue preparation and histological analysis

At embryonic day 18.5, anesthetized pregnant females were killed by cervical dislocation. Embryos were dissected in PBS, and the amniotic membrane was removed from the placenta. Placentae were fixed in in 4% paraformaldehyde for 24 h and stored in 70% ethanol until embedded in paraffin under standard procedures.

Placental sections (7 µm) were mounted on standard slides (Engelbrecht Medizin- und Labortechnik GmbH, Edermünde, Germany). For morphological analysis, sections were stained with Hematoxylin and Eosin (H&E).

### Morphometric analysis

Morphometric analysis of placentae and of placental compartments (labyrinth and spongiotrophoblast layer) was performed on nine serial sections of the central region of at least five placentae from each experimental group (control Ad0+PBS, *n*=5; AdsFlt+LPS, *n*=6) with an Axiophot model microscope (Carl Zeiss, Oberkochen, Germany) equipped with a DS-U1 camera and NIS-BR 3.1 software (Nikon, Düsseldorf, Germany). An auto-white balance correction was performed using ImageJ 1.51n (National Institutes of Health, Bethesda, MD, USA).

### RNA isolation and gene expression analysis

Total RNA from placentae was extracted with TriReagent (Life Technologies, Carlsbad, CA, USA). RNA quality and quantity was assessed with Nanodrop 2000c (Nanodrop Technologies, Wilmington, DE, USA). Complementary DNA (cDNA) synthesis was performed on 1 μg total RNA using M-MLV reverse transcriptase (Life Technologies), RNaseOUT (Life Technologies), random nonamers (Sigma-Aldrich). For quantitative real-time PCR (RT-qPCR), cDNA was amplified with TaqMan (Applied Biosystems) on a StepOnePlus™ Real-Time PCR System (Applied Biosystems). Primers used for RT-qPCR are listed in Table S2 (see also Kühnel et al., 2017). β-actin and *Gapdh* were used as housekeeping genes in all quantitative PCR analyses, and a standard curve method was used for quantification.

### Statistical analysis

Differences between groups were calculated with the Mann–Whitney *U*-test. Data are presented as median and interquartile ranges, if not stated otherwise. For all statistical tests, *P*<0.05 was considered significant. Pearson R correlation was used to check the association between selected parameters. Sample size was determined based on alpha=0.05, power 0.90, difference considered meaningful 20% and anticipated coefficient of variation 10. Data were analyzed using Prism 8 software (GraphPad) for Windows.

For metabolomics data, all the analyses were performed with MetaboAnalyst 3.0 (Xia and Wishart, 2016). For row-wise normalization, we chose to normalize with a reference sample (sample in the control with the least missing values); column-wise normalization was done by log2 transformation of the data. Univariate data analysis was performed using a volcano plot with fold-change threshold of 1.4 and *t*-test threshold of 0.1, as well as Mann–Whitney *U*-test. Multivariate data analysis was performed with PCA and PLS-DA in order to visualize the metabolic differences between controls and double-hit pre-eclampsia dams and fetuses. The PLS-DA model was assessed by its R2 and Q2 values to avoid the risk of overfitting, and cross-validation was performed with the leave-one-out cross-validation (LOOCV) model. The variable importance in the projection (VIP) scores higher than 1.0 were considered relevant for group discrimination (Jansson et al., 2009).

## Supplementary Material

Supplementary information
