# Supplementary material for: A double-hit pre-eclampsia model results in sex-specific growth restriction patterns
Source: Dis Model Mech. 2019 Feb 8;12(2):dmm035980. doi: 10.1242/dmm.035980 (PMC6398487; doi:10.1242/dmm.035980)
Supplement: Supplementary information [file dmm-12-035980-s1.pdf]

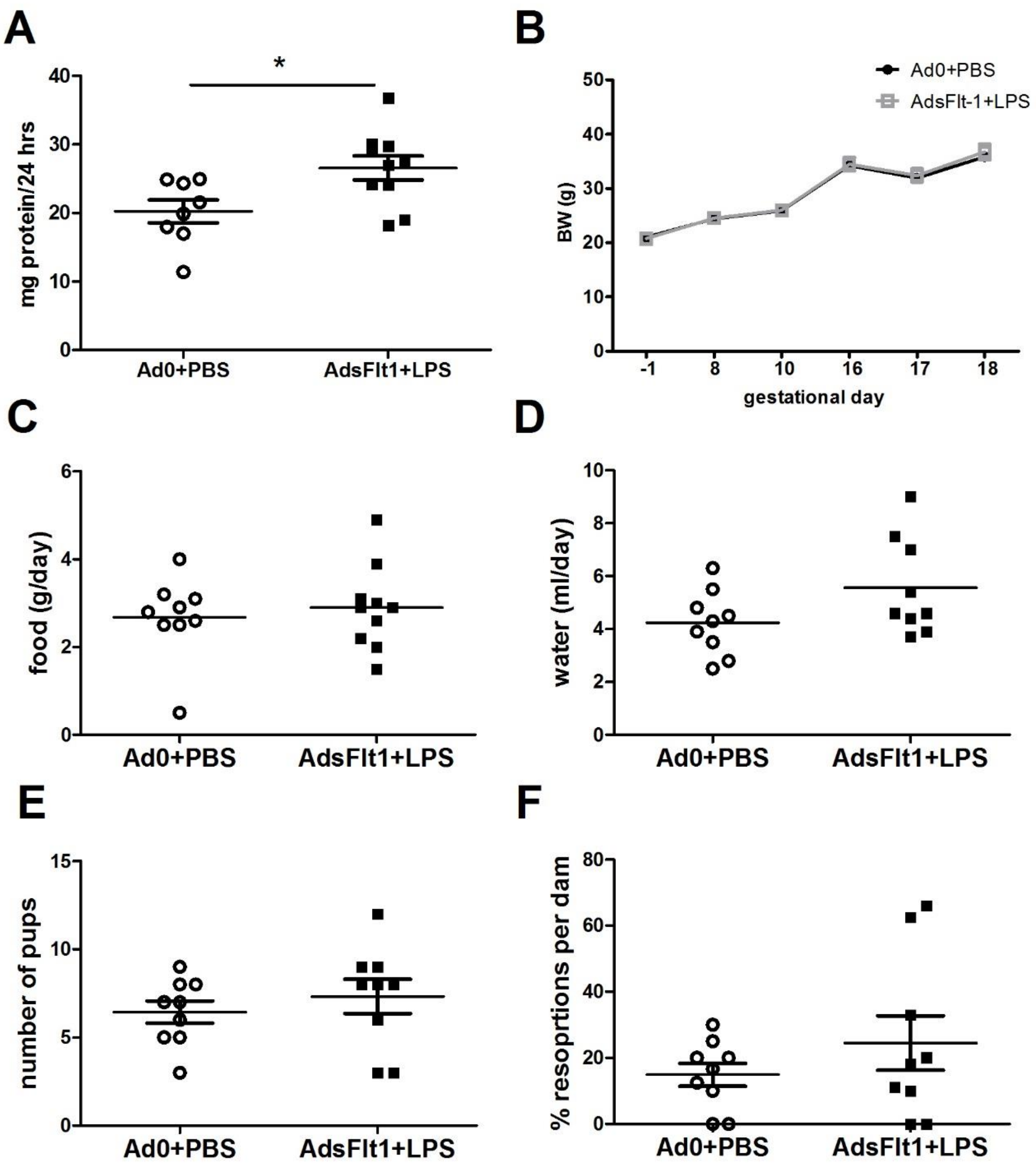

Figure S1. Maternal characteristics during double hit experimental preeclampsia (A) proteins in urine collected over 24 hours, (B) growth trajectories of pregnant dams, (C) food and (D) water consumption per day for pregnant dams, (D) number of pups and (E) and % of resorption per dam. Data given as median, *p<0.05.

Disease Models & Mechanisms • Supplementary information

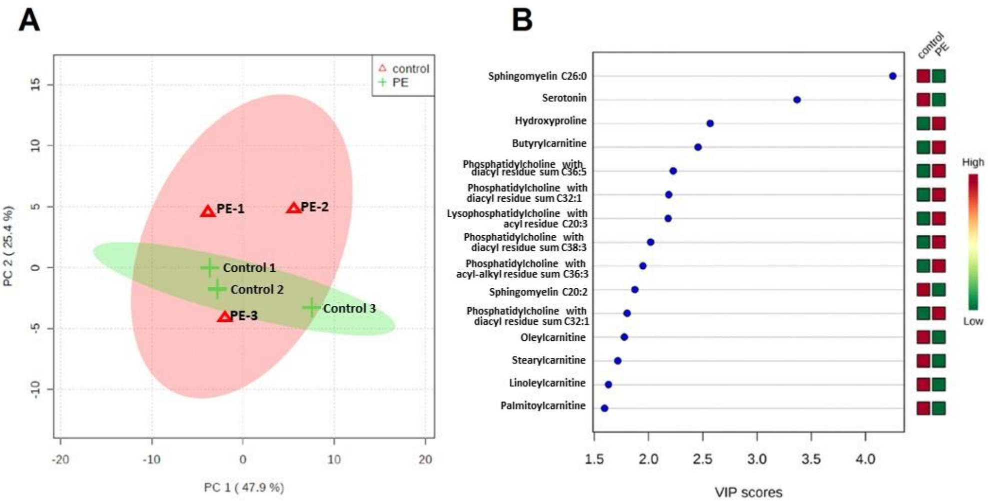

Figure S2. Metabolome characteristics of the dam (n=3), (A) PCA plot and, (B) VIP scores from supervised multivariate analysis of the dam metabolome.

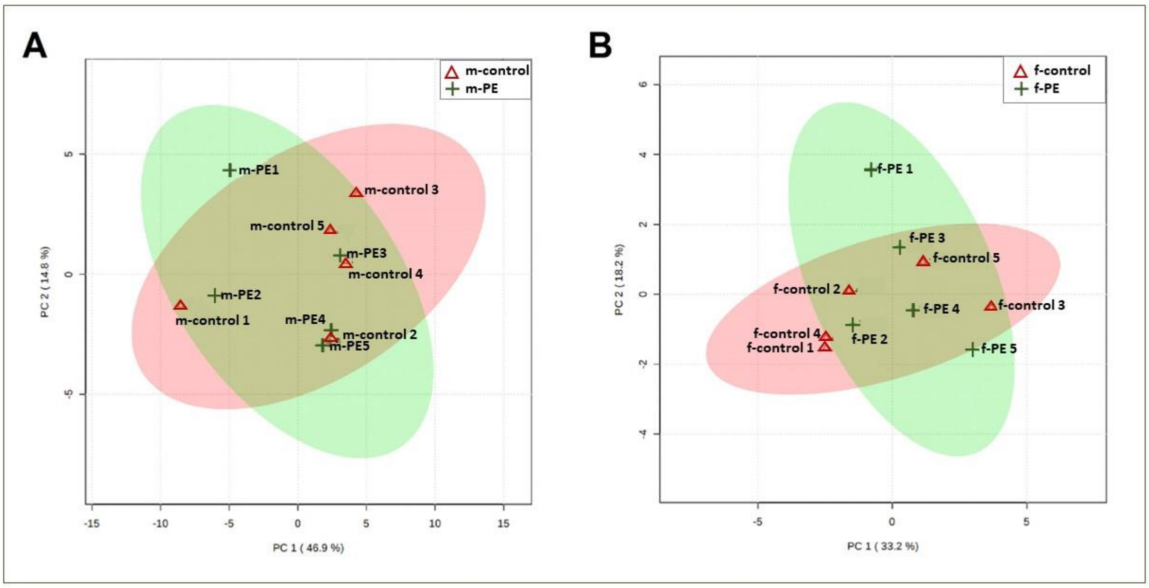

Figure S3. Metabolome characteristics of male and female fetuses exposed to double hit preeclampsia. (A) principal component analysis (PCA) plot for males and, (B) PCA plot for females.

Disease Models & Mechanisms • Supplementary information

Table S1. Metabolites with a low level of quantification (<LLOQ), that were excluded from the metabolomics analysis.

Excluded metabolites (conc <LLOQ)	
**Ac-Orn**	Acetylornithine
**Taurine**	
**PEA**	Phenylethylamine
**Nitro-Tyr**	Nitrotyrosine
**DOPA**	Dihydroxyphenylalanine
**Dopamine**	
**C3-OH**	Hydroxypropionylcarnitine
**C3:1**	Propenoylcarnitine
**C4:1**	Butenoylcarnitine
**C5-DC**	Glutaconylcarnitine
**C5-M-DC**	Methylglutarylcarnitine
**C5-OH**	Hydroxyisovalerylcarnitine
**C5:1**	Tiglylcarnitine
**C5:1-DC**	Glutaconylcarnitine
**C6**	Hexanoylcarnitine
**C6:1**	Hexenoylcarnitine
**C7-DC**	Pimelylcarnitine
**C8**	Octanoylcarnitine
**C9**	Nonanoylcarnitine
**C10**	Caprylcarnitine
**C10:1**	Decenoylcarnitine
**C10:2**	Decadienoylcarnitine
**C12**	Laurylcarnitine
**C12-DC**	Dodecanedioylcarnitine
**C12:1**	Dodecenoylcarnitine
**C14:1-OH**	Hydroxymyristoleylcarnitine
**C14:2**	Tetradecadienoylcarnitine
**C14:2-OH**	Hydroxytetradecadienoylcarnitine
**C16:1-OH**	Hydroxyhexadecenoylcarnitine
**C16:2**	Hexadecadienoylcarnitine
**C16:2-OH**	Hydroxyhexadecadienoylcarnitine
**C18:1-OH**	Octadecenoylcarnitine
**lysoPC a C14:0**	Lysophosphatidylcholine with acyl residue C14:0
**lysoPC a C28:0**	Lysophosphatidylcholine with acyl residue C28:0
**PC aa C26:0**	Phosphatidylcholine with diacyl residue sum C26:0
**PC aa C24:0**	Phosphatidylcholine with diacyl residue sum C24:0
**PC aa C30:2**	Phosphatidylcholine with diacyl residue sum C30:2
**PC aa C40:1**	Phosphatidylcholine with diacyl residue sum C40:1
**PC aa C42:0**	Phosphatidylcholine with diacyl residue sum C40:2

Disease Models & Mechanisms • Supplementary information

**PC ae C42:4**	Phosphatidylcholine with acyl-alkyl residue sum C42:4
**PC ae C42:0**	Phosphatidylcholine with acyl-alkyl residue sum C42:0
**PC ae C42:5**	Phosphatidylcholine with acyl-alkyl residue sum C42:5
**PC ae C44:4**	Phosphatidylcholine with acyl-alkyl residue sum C44:4
**PC ae C44:6**	Phosphatidylcholine with acyl-alkyl residue sum C44:6
**SM C22:3**	Sphingomyelin with acyl residue sum C22:3

Disease Models & Mechanisms • Supplementary information

Table S2. Primer sequences for quantitative RT-PCR on mouse placentae

Primer name	Forward sequence 5'-3'	Reverse sequence 5'-3'
mTOR	GACCTGAGCCGGCAGATTCC	GTGATCTGCGCAGTGTCGGA
Snat1	AGCACAGGCGACATTCTCATC	ACAGGTGGAACTTCGTCTTCTTG
Snat2	ACGAATGGGCTGTGGTATCTG	CCTAGATTTCTCAGCAGTGACAATG
Snat4	GGTCTCCCGGTCTAACCCTT	AAATTGGCTGTTCATGGCGT
Asct1	GGGCCATGTCATCCACGGAG	ATGAACACTGCGGCCACACA
4F2hc	CAGCGACCTGCTGTTGACCA	GCAGCAGCTGGTAGAGTCGG
Glut1	CAACGAGCATCTTCGAGAAGGC	CGTCCAGCTCGCTCTACAACAAAC
FatCD36	CCAGTGTATATGTAGGCTCATCCA	TGGCCTTACTTGGGATTGG
Fatp4	GGCTTCCCTGGTGTACTATGGAT	ACGATGTTTCCTGCTGAGTGGTA
Fatp6	GGCTTGAGGATGCCGCTTA	GTACTCTGGGCTCATGCTATGAAGT
Lpl	AATTTGCTTTCGATGTCTGAGAA	CAGAGTTTGACCGCCTTCC
Fabp1	GTGACTGAACTCAATGGAGACAC	GTAGACAATGTCGCCCAATGTCA
Fabp3	CATGAAGTCACTCGGTGTGG	TGCCATGAGTGAGAGTCAGG
Fabp4	AAGAAGTGGGAGTGGGCTTT	TCGACTTTCCATCCCACTTC
Fabp5	AGAGCACAGTGAAGACGAC	CATGACACACTCCACGATCA
